# Uniportal VATS right superior lobectomy: management of pulmonary vein variation: a case report

**DOI:** 10.1186/s13019-020-1088-3

**Published:** 2020-02-27

**Authors:** Zhao Wang, Yungang Sun, Qiang Zhang, Feng Shao

**Affiliations:** Department of Thoracic Surgery, Nanjing Chest Hospital, Nanjing Brain Hospital Affiliated to Nanjing Medical University, Nanjing, 210029 China

**Keywords:** Variation, Pulmonary vein, VATS, Surgery, Lung cancer

## Abstract

**Background:**

Although there are lots of variations of pulmonary veins including dangerous type that could cause serious complications during surgery, limited information has been reported about these variations. We have experienced an extremely rare anomaly of the right superior pulmonary vein during right superior lobectomy. We used a technique called “non fissure” to manage the right superior pulmonary vein, and the results were satisfactory.

**Case presentation:**

A 66-year-old woman with lung nodules visited our hospital. Chest computed tomography revealed multiple ground glass nodules in the right lung, the main pulmonary nodule was 11 mm in diameter and presented mixed density. The patient had a previous history of rectal cancer surgery. Contrast-enhanced three-dimensional computed tomography showed that the right superior pulmonary vein abnormally ran between the pulmonary artery trunk and the right main bronchus. We performed a right superior lobectomy and lymph node sampling by uniportal video-assisted thoracoscopic surgery. The pathological findings showed microinvasive adenocarcinoma with no lymphatic metastasis. She was discharged 7 days after surgery without any surgical complications.

**Conclusions:**

Although the variation of pulmonary vein is uncommon, it is dangerous to misidentify in the operation. Preoperative three-dimensional computed tomography is useful for avoiding unexpected bleeding. The technique “no fissure” might be a useful way to manage the variation of pulmonary vein.

## Background

Anatomic lobectomy is the standard surgical procedure for the treatment of lung cancer. The pulmonary arteries, veins, and bronchi of the target lobe need to be dissociated and severed separately. Vascular variations sometimes make vascular anatomy difficult. Although pulmonary vein variation is uncommon, some had been reported about these variations. We report a rare case about anatomic variation of the right superior pulmonary vein (SPV). Chest three-dimensional computed tomography (3D-CT) found the patient’s right SPV ran abnormally between the right main pulmonary artery trunk (PAT) and the right main bronchus. The variation had the potential to cause serious unexpected bleeding during surgery. During the operation, we used a technique called “non fissure” to manage the right SPV, and the results were satisfactory.

## Case presentation

**Fig. 1:**
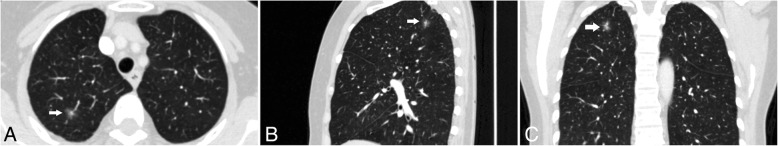
Chest computed tomography revealed the main pulmonary nodule located in the right upper lobe was 11 mm in diameter and presented mixed density

**Fig. 2:**
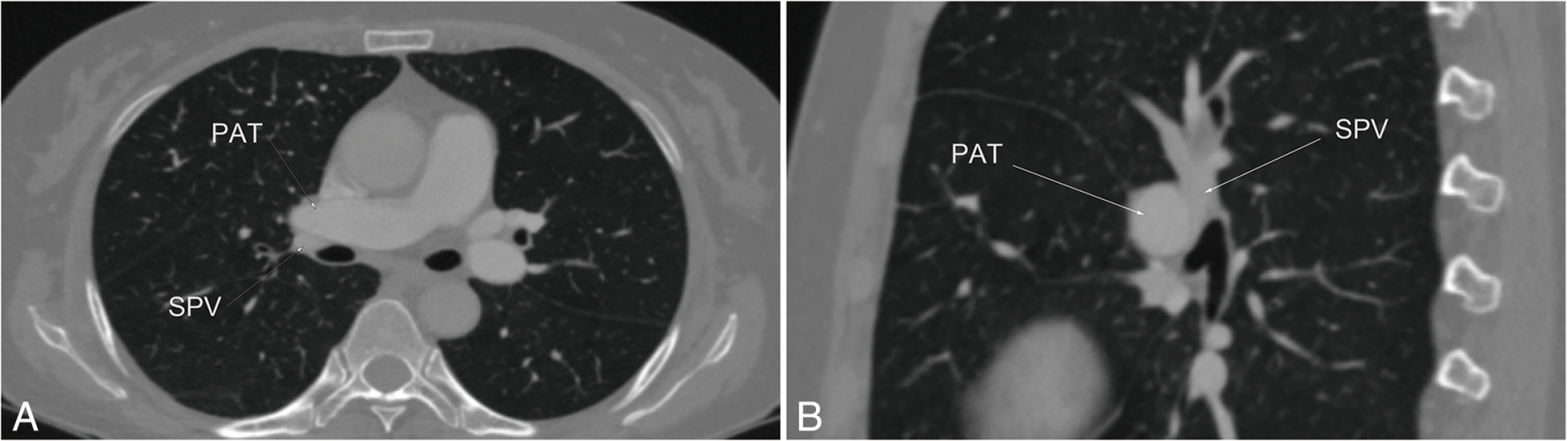
Contrast-enhanced three-dimensional computed tomography showed that the superior pulmonary vein abnormally ran between the pulmonary artery trunk and the right main bronchus

**Fig. 3:**
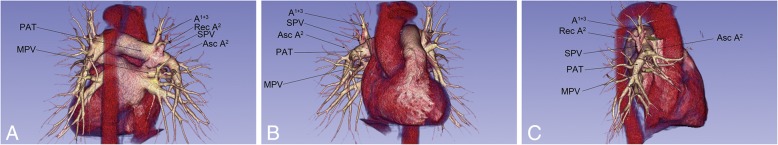
Contrast-enhanced three-dimensional computed tomography reconstruction (volume rendering) showed the variation that the superior pulmonary vein abnormally ran between the pulmonary artery trunk and the right main bronchus

**Fig. 4:**
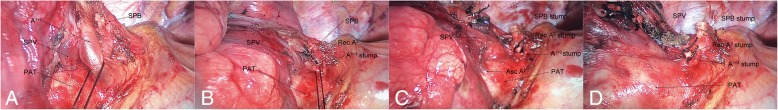
The operative findings confirmed that the superior pulmonary vein abnormally ran between the pulmonary artery trunk and the right main bronchus

A 66-year-old woman with lung nodules visited our hospital. Chest CT revealed multiple ground glass nodules in the right lung, the main pulmonary nodule was 11 mm in diameter and presented mixed density (Fig. 1). The patient had a previous history of rectal cancer surgery. Contrast-enhanced 3D-CT showed that the SPV ran abnormally between the PAT and the right main bronchus (Fig. 2 and Fig. 3). A systemic CT examination revealed no other tumors. We performed a right superior lobectomy and lymph node sampling by uniportal video-assisted thoracoscopic surgery (VATS) for the patient. The operative findings confirmed the truth that the SPV abnormally ran between PAT and the right main bronchus (Fig. 4). It is difficult to dissociate the right SPV alone. We removed the right SPV together with the pulmonary fissure, which is called the “no fissure” technique. The postoperative course was uncomplicated. The pathological findings showed microinvasive adenocarcinoma with no lymphatic metastasis. She was discharged 7 days after surgery without any surgical complications.

## Discussion

There are many variants of PVs, and few of these variants have been reported in the literature. Fourdrain et al. retrospectively reviewed the CT images and medical records of 100 patients. Anatomic variations of PVs were found in 36 patients (36%), and right-sided anatomical variations were more frequent than on the left sided (25% vs. 11%).The most common variations were three independent PVs on the right side (16%), while single PV (8%) on the left side, including surgical conversion (21%) [1]. Kawasaki et al. reported a rare anomalous SPV drained into the azygos vein along the superior vena cava in 2017 [2]. Aragaki et al. reported a rare anomalous left V2 drained into the inferior PV in 2017 [3]. Low et al. reported an aberrant left SPV drained into the innominate vein in 2018 [4]. Asouhidou et al. reported a case that the common trunk of the left superior and inferior PV was misidentified as an inferior PV, and it was transected during surgery in 2017 [5]. Shapiro et al. reported a case that the left upper lobe PV was not entering the atrium, but instead superiorly up into the brachiocephalic vein in 2014 [6]. Sumitomo et al. reported a case of the same vascular variation as this case in 2016, that is, the right SPV ran behind the PAT and in front of the right main bronchus [7]. Unlike in this case, there was also a posterior segmental bronchial variation.

In traditional right superior lobectomy, the arteries and veins of the right upper lobe need to be isolated separately, including the SPV, posterior segmental artery, anterior and apical segmental trunk. When the position of SPV changed, the traditional operation procedure would cause the operation more difficult, leading to blood vessel rupture and massive hemorrhage. For thoracic surgeons, abnormal PVs could lead to miscalculation of anatomy, increasing the difficulty and risk of surgery. Uniportal VATS lobectomy has been recognized as a safe and effective surgical procedure. Compared with three portal VATS, the technique might be more difficult. In this case, the patient’s right SPV was located behind the pulmonary trunk and was difficult to dissect separately. We removed the right SPV together with the pulmonary fissure, which was called the “no fissure” technique. Lin et al. reported a surgical technique that simplifies synchronous disconnection of pulmonary arteries and veins for right superior lobectomy [8]. In uniportal VATS, the exposure of the operative field is different from that of three-portal VATS, and the traction direction of the pulmonary lobe is limited as common, so the release of the pulmonary hilus is particularly important. Compared with that technique, we prioritized managed posterior and anterior segmental artery to reduce the risk of vascular rupture due to excessive traction of the pulmonary lobe during uniportal VATS. We extended this method to all right upper lobectomies and found that it is a practical and simple surgical method. It can speed up surgery and reduce the risk of blood vessel rupture during surgery.

## Conclusion

Although the variation of PV is uncommon, it is dangerous to misidentify in the operation. Preoperative 3D-CT is useful for avoiding unexpected bleeding. The technique “no fissure” might be a useful way to manage the variation of pulmonary vein.

## Data Availability

Please contact author for request.

## Abbreviations

3D-CT: Three-dimensional computed tomography
PAT: Pulmonary artery trunk
SPV: Superior pulmonary vein
VATS: Video-assisted thoracoscopic surgery
